# Retrograde Intramedullary Nailing for Distal Femur Fractures: A Prospective Study of Functional Outcomes, Complications, and Union Rates

**DOI:** 10.7759/cureus.82139

**Published:** 2025-04-12

**Authors:** Raj Kishore, Mahfooj Alam, Nabeel Thahseen, Bavithiran CM, Kishore Vellingiri

**Affiliations:** 1 Department of Orthopaedics, Indira Medical College and Hospitals, Tiruvallur, IND; 2 Department of Orthopaedics, Tirunelveli Medical College, Tirunelveli, IND; 3 Department of Orthopaedics, Vinayaka Mission’s Medical College, Karaikal, IND; 4 Orthopaedics, Sri Devaraj Urs Academy of Higher Education and Research, Kolar, IND

**Keywords:** complications, distal femur fracture, functional outcomes, minimal invasion, modified mize score, retrograde intramedullary nailing, union rate

## Abstract

Background

Distal third femur fractures, especially those with intra-articular extension, are challenging to manage and often lead to complications. Retrograde intramedullary nailing (RIN) has emerged as a minimally invasive alternative to traditional plating, offering advantages such as reduced soft tissue trauma, lower infection rates, and faster healing. This study aimed to evaluate the efficacy of RIN in managing distal femur fractures with associated soft tissue damage.

Methods

A prospective analysis was conducted involving 39 patients with distal femur fractures treated with retrograde nailing at Tirunelveli Government Medical College and Hospital between 2023 and 2025. Inclusion criteria included patients aged 20-70 years with AO type A1, A2, A3, C1, and C2 fractures, as well as Gustilo-Anderson type I and II fractures. Exclusion criteria included pathological fractures, periprosthetic fractures, and vascular injuries. Functional and radiological outcomes were assessed using the Modified Mize criteria and the Knee Society Scoring System.

Results

Functional outcomes were rated as excellent in 64.10% of patients, good in 30.77%, fair in 2.56%, and poor in 2.56%. The mean operative time was 94.36 ± 14.64 minutes, and the mean blood loss was 220 ± 47.60 ml for open nailing and 178.18 ± 38.28 ml for closed nailing. Union was achieved in 94.9% of cases, with one case of delayed union and one case of non-union requiring revision surgery. Knee stiffness was the most common complication observed, with an average knee flexion of 123.72 degrees. No infections or implant failures were reported.

Conclusion

RIN is an effective treatment for distal femur fractures, particularly in patients with soft tissue damage. It offers high union rates, low complication rates, and improved functional outcomes. However, surgeons should be mindful of potential complications such as knee stiffness and ensure adherence to postoperative rehabilitation protocols. This study contributes valuable insights into the use of intramedullary femur fixation for distal third femur fractures. Further multi-centre studies with larger sample sizes are recommended to validate these findings.

## Introduction

Distal third femur fractures are among the most common orthopedic injuries, particularly in high-energy trauma scenarios such as motor vehicle accidents or falls from significant heights [1]. These fractures are often complex, especially when accompanied by intra-articular extension, which significantly elevates the risk of complications such as malunion, non-union, and post-traumatic arthritis [2]. While traditional fixation methods, such as plates and screws, have been widely used, they are associated with higher rates of infection, delayed wound healing, and soft tissue complications due to the extensive surgical exposure required [3].

In recent years, retrograde intramedullary nailing (RIN) has emerged as a preferred treatment option for distal femur fractures, particularly in cases with soft tissue damage. This minimally invasive technique offers several advantages, including reduced soft tissue trauma, lower infection rates, and faster healing compared to traditional plating methods [4,5]. The intramedullary position of the nail provides biomechanical stability, reducing the risk of varus and valgus malalignment - a common issue with lateral plating [6]. Moreover, retrograde nailing facilitates early mobilization and weight-bearing, which are critical for functional recovery and minimizing postoperative complications such as knee stiffness [7].

Despite these advantages, the use of retrograde nailing in distal femur fractures remains a topic of debate, particularly in cases with severe comminution or intra-articular involvement. Some studies have reported challenges such as posterior angulation, knee joint cartilage damage, and the risk of early osteoarthritis due to the proximity of the nail to the knee joint [8]. However, recent advancements in implant design and surgical techniques have addressed many of these concerns, making retrograde nailing a viable option for a broader range of fracture patterns [9].

This study aims to evaluate the efficacy of RIN in the management of distal third femur fractures, with a focus on cases involving soft tissue damage. Specifically, we investigate the outcomes of a one-stage surgical procedure in patients for whom plate and screw fixation was deemed unsuitable due to the extent of soft tissue injury. We hypothesized that retrograde nailing effectively reduces complications associated with concurrent soft tissue damage, offering a safer and more reliable alternative to traditional plating methods.

## Materials and methods

This prospective analysis evaluated the functional and radiological outcomes of distal femur fractures treated with retrograde nailing. It included 39 patients admitted to the Department of Orthopedics at Tirunelveli Government Medical College and Hospital between 2023 and 2025. All patients presented with distal femur fractures and were aged between 20 and 70 years. Fractures included in the study were classified as AO types A1, A2, A3, C1, and C2, as well as Gustilo-Anderson type I and II open fractures. However, patients with pathological femoral fractures, periprosthetic supracondylar fractures, stiff knees, AO types B and C3 fractures, or fractures with vascular injuries were excluded from the study.

Operating procedure

**Figure 1:**
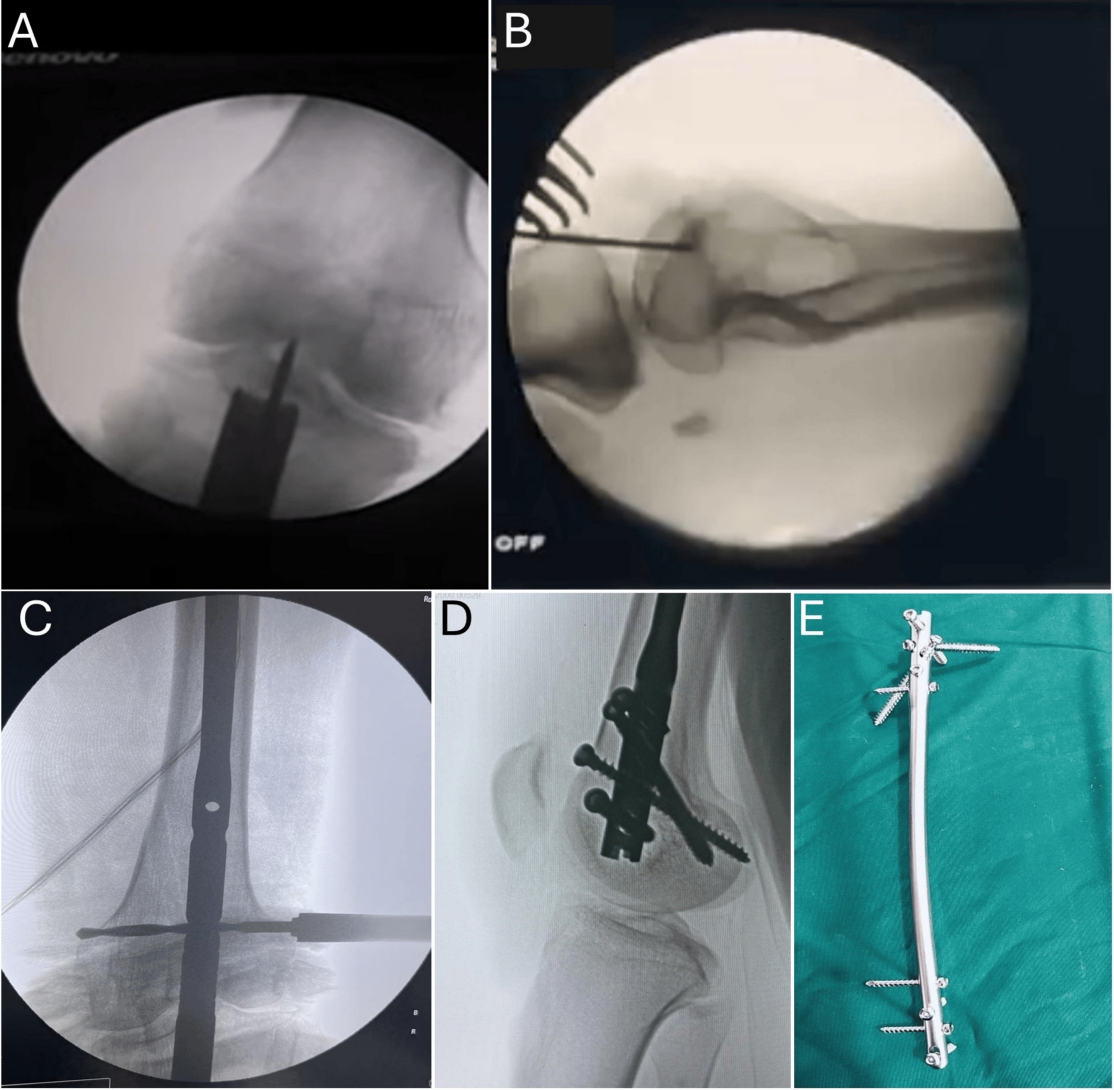
Intraoperative fluoroscopic images A, B, and C: Entry made under fluoroscopic guidance. D: Distal locking done using interlocking bolts. E: Multifunctional retrograde femoral nail (MRFN)

To facilitate fracture reduction, patients were positioned supine on a radiolucent table with a rolled sterile bump placed precisely at fracture apex and the knee flexed at 30° [7]. This position improves access to the intercondylar notch for nail entry, maintains reduction during guidewire placement and reaming, and also allows easier assessment of rotational alignment under fluoroscopy. This positioning was further supported by the use of a knee-spanning femoral distractor and joysticks inserted into the distal fragment. All 39 patients underwent fixation using the Multifunctional Retrograde Femur Nail (MRFN, Nebula®, Rajkot, India) under spinal anesthesia. A 3-cm skin incision was made over the patellar tendon while the knee remained flexed at 30°. Under fluoroscopic guidance, the entry point was established at the intercondylar notch, positioned just anterior to the Blumensaat line and aligned with the femoral shaft axis (Figure 1). Sequential reaming was performed, followed by the insertion of an appropriately sized nail. In cases where the fracture extended intra-articularly, screws were used to stabilize the joint before nailing. The nail lengths ranged from 200-280 mm and had diameters of 10-12 mm.

Immediate postoperative knee mobilization and quadriceps strengthening exercises were initiated to promote recovery. Patients were advised to avoid weight-bearing initially, with partial weight-bearing permitted at four weeks post-surgery. Full weight-bearing was allowed after six weeks, provided there was clinical and radiological evidence of bone union. Union was assessed by radiographic bridging callus in ≥3 cortices and clinically by pain-free weight-bearing. Data collected from patients included age, gender, the side of the fracture, and the fracture type according to the AO classification system. Complications, time to union, and postoperative reduction quality were assessed using the Modified Mize criteria and the Knee Society Scoring System [10]. Statistical analysis was performed to evaluate the outcomes.

## Results

Of the 39 patients included in the study, 25 (64.10%) achieved excellent functional outcomes, while 12 (30.77%) had good outcomes. One patient (2.56%) demonstrated fair results, and another (2.56%) experienced poor outcomes. The mean age of the participants was 39.5 years, with a significant male predominance: 28 individuals (71.79%) were male, and 11 (28.21%) were female, as detailed in Table 1.

**Table 1:** Demographic and injury-related characteristics of the study population (N=39) RTA: road traffic accident

Parameter	N (%)
Gender	
Male	28 (71.79%)
Female	11 (28.21%)
Age group, years	
20-40	20 (51.28%)
41-60	16 (41.03%)
>61 years	3 (7.69%)
Side	
Right	25 (64.10%)
Left	14 (35.90%)
Mode of injury	
RTA	34 (87.18%)
Accidental fall	5 (12.82%)

In terms of treatment methods, 12 patients (30.77%) presented with intra-articular fracture extensions, which were initially stabilized using cancellous screws. All patients were subsequently treated with MRFN. Functional and radiological outcomes were assessed using the Knee Society Scoring System and the Modified Mize criteria. According to the Modified Mize criteria, an excellent reduction was defined as a loss of knee flexion of less than 10 degrees, full extension, absence of varus, valgus, or rotational deformities, no pain, and perfect joint alignment. In contrast, a poor reduction was characterized by knee flexion of less than 90 degrees, varus deformity exceeding 10 degrees, valgus deformity greater than 15 degrees, joint incongruity, and persistent pain, regardless of radiological findings.

Road traffic accidents (RTAs) were the most common mode of injury, accounting for 87.18% of cases. RTAs were particularly prevalent among young males, while trivial trauma, likely due to osteoporosis, was more common among older females. In terms of limb involvement, the left limb was fractured in 14 patients (35.90%), whereas the right limb was affected in 25 patients (64.10%). The majority of patients (n=34, 87.18%) sustained closed injuries. Open fractures, which involved soft tissue damage, were classified according to the Gustilo-Anderson system. These fractures, often resulting from high-energy trauma, were frequently associated with additional injuries such as head injuries, ipsilateral both-bone leg fractures, tibial condyle fractures, and both-bone forearm fractures.

**Figure 2:**
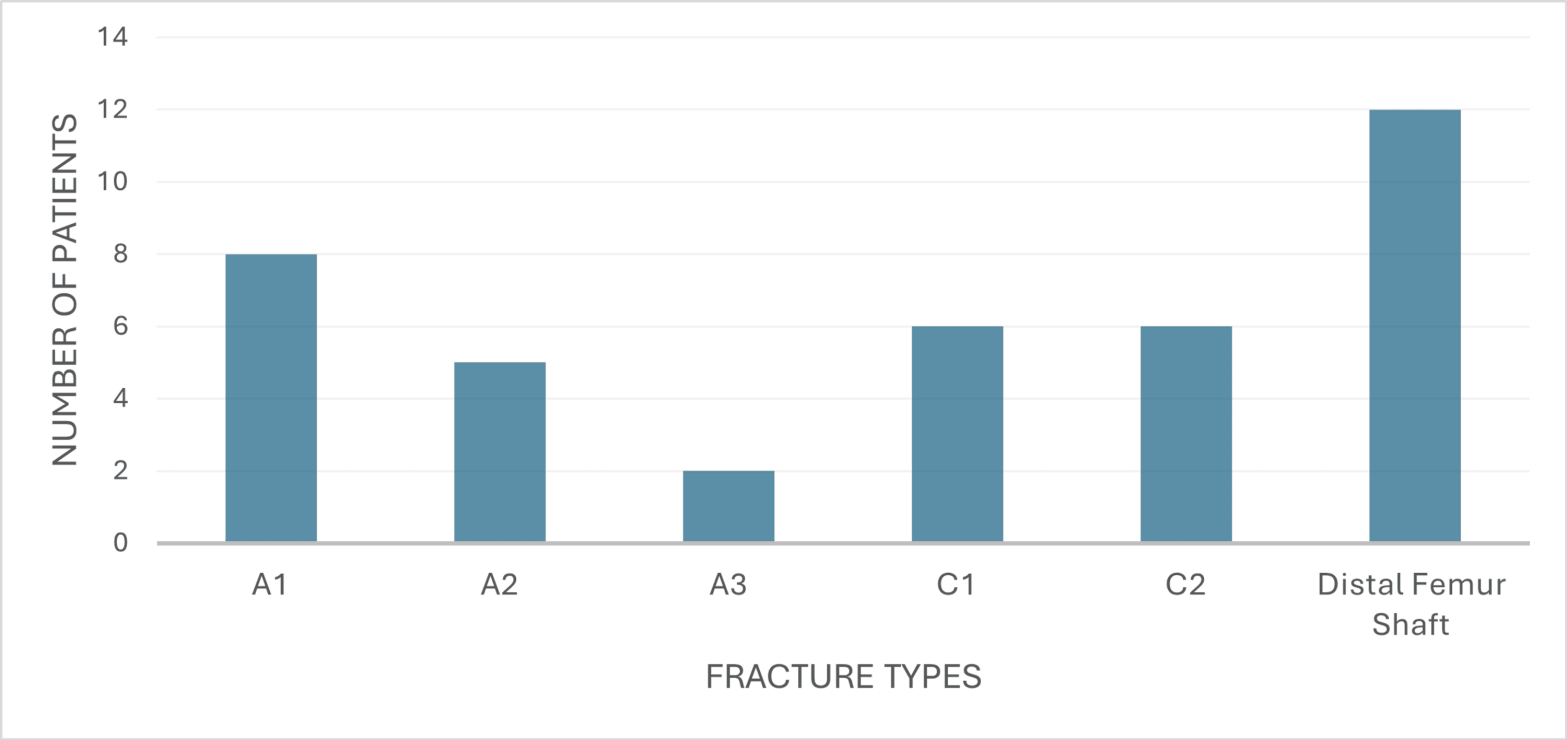
Distribution of fracture types

The cases operated on were classified according to the AO system as types A1, A2, A3, C1, C2, and distal femur shaft fractures, as illustrated in Figure 2 and Table 2. Closed reduction was performed in 33 cases (84.62%), while open reduction was required in six cases (15.38%). The average blood loss during the procedure was 220 ± 47.60 ml for open nailing and 178.18 ± 38.28 ml for closed nailing, as detailed in Table 3. Surgical intervention was delayed until soft tissue stabilization was achieved, with a mean interval of five (range: 2-10) days. No temporary ex-fix was used.

**Table 2:** Distribution of fracture types (N=39)

Type of fracture	N (%)
A1	8 (20.51%)
A2	5 (12.82%)
A3	2 (5.12%)
C1	6 (15.38%)
C2	6 (15.38%)
Distal femur shaft	12 (30.77%)

**Table 3:** Methods of nailing among patients and its corresponding mean blood loss (N=39)

Methods of nailing	N (%)	Mean blood loss (ml)
Open	6 (15.38%)	220 ± 47.60
Closed	33 (84.62%)	178.18 ± 38.28

**Figure 3:**
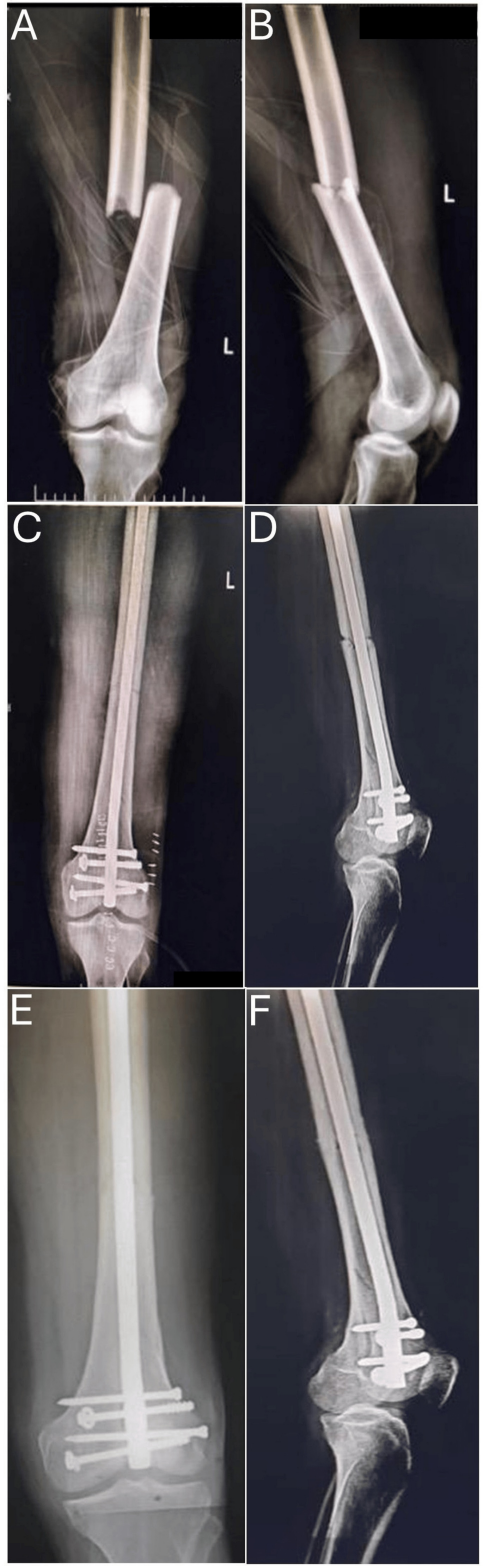
Multifunctional retrograde femoral nail fixation - image 1 (A) Preoperative anteroposterior radiograph of distal femur fracture. (B) Preoperative lateral radiograph of distal femur fracture. (C) Postoperative (immediate) anteroposterior radiograph. (D) Postoperative (immediate) lateral radiograph. (E) Postoperative (one-year follow-up) anteroposterior radiograph. (D) Postoperative (one-year follow-up) lateral radiograph

**Figure 4:**
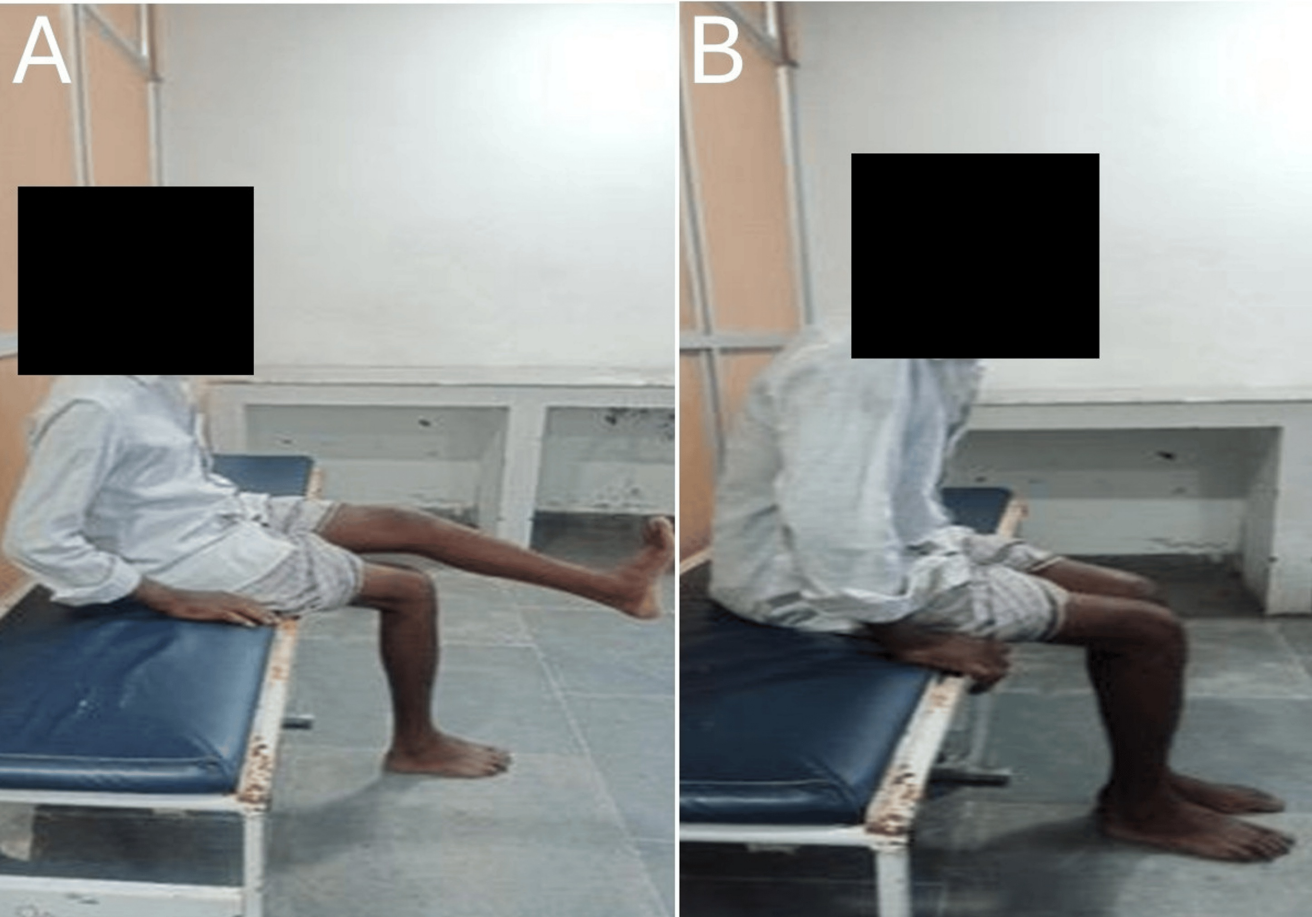
Postoperative knee ROM with flexion >90 degrees without stiffness ROM: range of motion

The mean operative time for the procedure was 94.36 ± 14.64 minutes. Radiation exposure during surgery was measured by the number of C-arm shots taken, with a mean of 30 ± 6.2, ranging from 18-42 shots per procedure. Postoperatively, patients were followed up at six weeks, three months, six months, and one year. During each follow-up visit, a comprehensive evaluation was conducted to assess quadriceps function, knee range of motion (ROM), and any potential complications, as illustrated in Figures 3, 4.

X-rays were routinely taken during follow-up visits to assess fracture union and identify any signs of failure. The mean follow-up duration was 37 weeks, with an average union time of 22 weeks. Among the 39 patients, one case of delayed union and one case of non-union were observed. The non-union was successfully managed with revision surgery, ultimately achieving union without further complications. Knee stiffness emerged as the most frequently encountered complication, with an average knee flexion of 123.72 degrees recorded in the study, as detailed in Table 4.

**Table 4:** Postoperative knee ROM ROM: range of motion

Knee ROM flexion	N (%)
>90 degrees	34 (87.18%)
<90 degrees	5 (12.82%)

Several factors contributed to the observed complications, including delays in surgery, the complexity of the fracture pattern, the stability of fixation, and a lack of adherence to intensive physiotherapy. Among the patients, three individuals had knee flexion of 90 degrees or less. Of these, two had intra-articular fractures, and one presented with grade four osteoarthritis of the knee. Notably, all three patients were non-compliant with postoperative rehabilitation exercises. Moderate knee pain was reported in 15.38% of cases, though it was effectively managed with analgesics. No instances of implant failure were observed.

## Discussion

**Figure 5:**
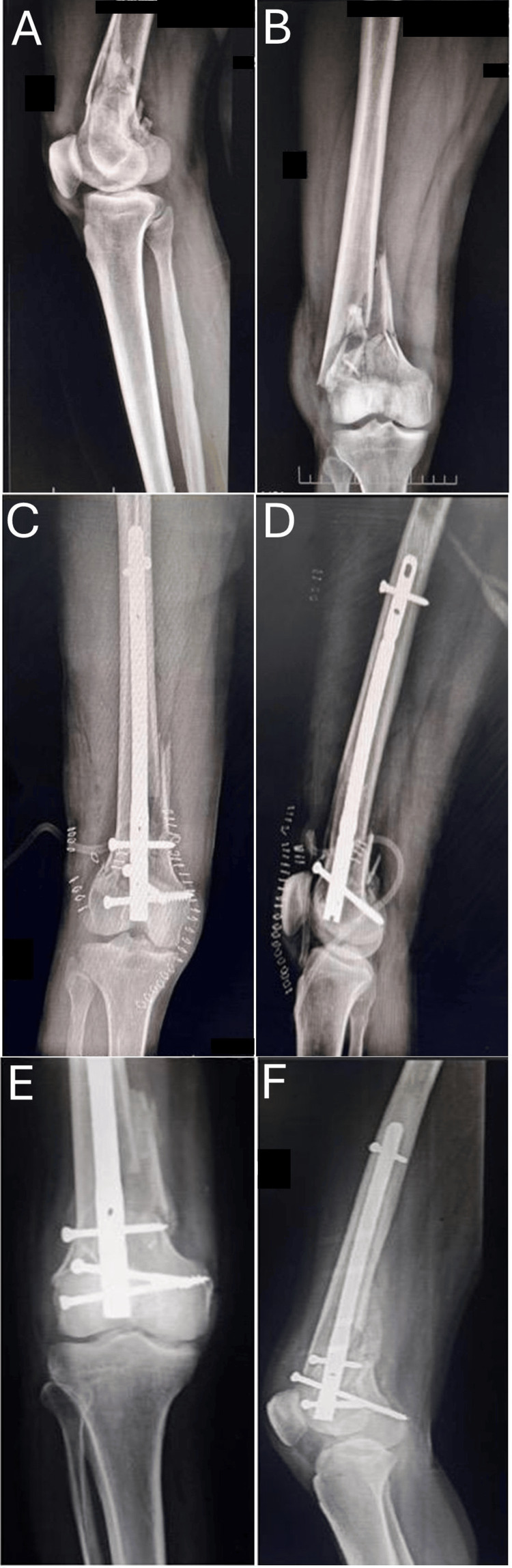
Multifunctional retrograde femoral nail fixation - image 2 (A) Preoperative anteroposterior radiograph of AO C2 type fracture. (B) Preoperative lateral radiograph of AO C2 type fracture. (C) Postoperative (immediate) anteroposterior radiograph. (D) Postoperative (immediate) lateral radiograph. (E) Postoperative (one-year follow-up) anteroposterior radiograph. (D) Postoperative (one-year follow-up) lateral radiograph

**Figure 6:**
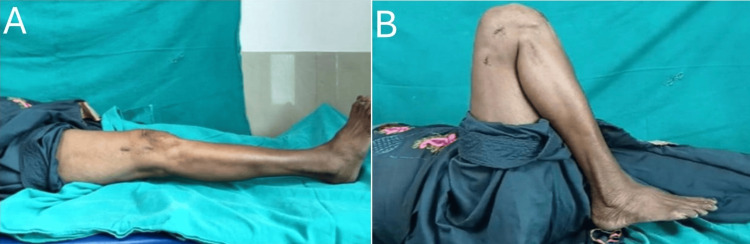
Postoperative knee ROM without extensor lag or stiffness (achieved ROM >90-degree pain free) ROM: range of motion

Despite significant advancements in modern fixation techniques, fractures of the distal femur continue to pose challenges, often leading to persistent disability and suboptimal outcomes [8]. In younger individuals, these injuries are typically caused by high-energy trauma, such as motor vehicle accidents, which can also result in open fractures. In contrast, elderly patients with osteoporotic bones are more likely to sustain such fractures from low-energy events, such as falls. Regardless of the mechanism, most distal femur fractures tend to be comminuted, adding to the complexity of their management. The treatment of distal femur fractures demands a precise and skillful approach to achieve successful outcomes. While newer, biologically favorable fixation methods have helped reduce complication rates, these fractures remain a significant challenge for orthopedic surgeons, as illustrated in Figures 5, 6.

Historically, open reduction and internal fixation (ORIF) has been the treatment of choice for distal femur fractures, emphasizing accurate reduction and stable fixation to prevent complications such as non-union, functional impairment, deformity, and the need for additional surgeries. Despite advancements in surgical techniques and implant design, non-union rates and the requirement for secondary procedures remain significant concerns in managing these fractures [8]. Over time, treatment approaches have evolved from using condylar screws and angled blade plates to more modern techniques such as the Less Invasive Stabilization System (LISS) and RIN [9].

Retrograde nailing, in particular, involves the indirect reduction of the metaphyseal fracture component while providing relative stability [2]. This technique was developed to address some of the challenges associated with traditional methods of treating distal femur fractures. According to Henry et al., retrograde nails offer a notable advantage over laterally placed devices due to their intramedullary position, which minimizes the lever arm effect and reduces the risk of varus and valgus angulation [4]. However, the technique is not without limitations, including the potential for posterior angulation, damage to knee joint cartilage, an increased risk of early osteoarthritis, and the intra-articular effects caused by reaming debris.

Numerous studies suggest that intramedullary nailing results in higher union rates compared to other fixation methods. The rigidity of the fixation construct plays a critical role in determining fracture stability, which directly influences the healing process. For instance, Gill et al. reported a 90% union rate with retrograde nailing techniques [11], while Gao et al. observed a 94.1% union rate, attributing faster healing to the release of intramedullary contents at the fracture site during reaming [12]. Intramedullary stabilization, particularly when combined with a longer working length, appears to significantly contribute to faster bone union. In our study, only one case of non-union was observed, which was successfully treated with open reduction, internal fixation using a plate, and bone grafting. Additionally, one case of delayed union was noted, which eventually healed with prolonged follow-up.

The mean operative time in our study was 94.36 ± 14.64 minutes, with variations depending on factors such as surgeon experience, implant availability, and operating theatre standards. Comparatively, Gill et al. noted a mean operative time of 102.3 ± 20.6 minutes [11]. The average knee range of motion (ROM) in our study was 123.7 degrees, higher than the 107.0 degrees reported by Gill et al. [11] and the 116 degrees reported by Yadav et al. [13]. Notably, no cases of infection were observed in our study, consistent with findings by Ahmed et al. and Yadav et al., who reported significantly lower infection rates with retrograde nailing compared to plating [13,14]. Streubel et al. highlighted that elderly patients with distal femur fractures are at higher risk of mortality, emphasizing the importance of minimally invasive techniques like retrograde nailing to reduce surgical trauma and improve recovery [15].

**Figure 7:**
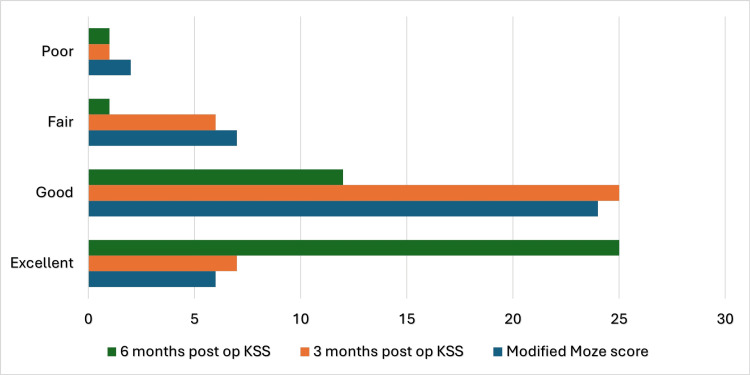
Functional outcomes in our study at 3 months, 6 months, and Modified Mize scores KSS: Knee Society Score

The union rate of 94.9% in our study was not significantly different from the expected rate of 90% (p=0.308). However, there was a significant improvement in the Knee Society Score between three and six months postoperatively (p<0.001). Similarly, the Modified Mize score showed a statistically significant difference from the expected equal distribution (p<0.001), as illustrated in Figure 7 and Table 5. While complications such as guidewire breakage, bending, or misplacement of locking screws have been reported in the literature, none were observed in our study. Nevertheless, these complications remain potential risks associated with femoral nailing.

**Table 5:** Functional outcomes in our study at 3 months, 6 months, and Modified Mize scores KSS: Knee Society Score

Results	KSS 3 months post-op, n (%)	KSS 6 months post-op, n (%)	Modified Mize score, n (%)
Excellent	7 (17.95%)	25 (64.10%)	6 (15.38%)
Good	25 (64.10%)	12 (30.77%)	24 (61.53%)
Fair	6 (15.38%)	1 (2.56%)	7 (17.95%)
Poor	1 (2.56%)	1 (2.56%)	2 (5.12%)

Our study has several limitations, including a relatively small sample size, the absence of a control group, and a single-center design. Additionally, the lack of evaluation of postoperative functional outcomes and the heterogeneity in injury mechanisms and fracture types may limit the generalizability of our findings. Despite these limitations, the results provide valuable insights into the efficacy of retrograde nailing for distal femur fractures and underscore the need for further research to address these constraints.

## Conclusions

RIN has proven to be a highly effective treatment for distal femur fractures, particularly in cases involving soft tissue injury. The study highlights the procedure's ability to achieve high union rates, minimize complications, and improve functional outcomes. Surgeons should prioritize postoperative rehabilitation to address potential issues, such as knee stiffness, and ensure optimal recovery. While the findings are promising, the study's limitations, including its small sample size and single-center design, underscore the need for future research. Multi-center, randomized controlled trials with larger patient cohorts and long-term follow-up are essential to further validate these results and explore the durability of outcomes.
